# Non-fullerene acceptor organic photovoltaics with intrinsic operational lifetimes over 30 years

**DOI:** 10.1038/s41467-021-25718-w

**Published:** 2021-09-14

**Authors:** Yongxi Li, Xiaheng Huang, Kan Ding, Hafiz K. M. Sheriff, Long Ye, Haoran Liu, Chang-Zhi Li, Harald Ade, Stephen R. Forrest

**Affiliations:** 1 Departments of Electrical Engineering, Material Science and Engineering, and Physics, University of Michigan, Ann Arbor, MI 48109 USA; 2 Applied Physics Program, University of Michigan, Ann Arbor, MI 48109 USA; 3 Department of Physics and Organic and Carbon Electronics Laboratories (ORaCEL), North Carolina State University, Raleigh, NC 27695 USA; 4 School of Materials Science and Engineering and Tianjin Key Laboratory of Molecular Optoelectronic Sciences, Tianjin University, 300072 Tianjin, China; 5 State Key Laboratory of Silicon Materials, MOE Key Laboratory of Macromolecular Synthesis and Functionalization, Department of Polymer Science and Engineering, Zhejiang University, 310027 Hangzhou, China

**Keywords:** Solar energy, Electrical and electronic engineering

## Abstract

Organic photovoltaic cells (OPVs) have the potential of becoming a productive renewable energy technology if the requirements of low cost, high efficiency and prolonged lifetime are simultaneously fulfilled. So far, the remaining unfulfilled promise of this technology is its inadequate operational lifetime. Here, we demonstrate that the instability of NFA solar cells arises primarily from chemical changes at organic/inorganic interfaces bounding the bulk heterojunction active region. Encapsulated devices stabilized by additional protective buffer layers as well as the integration of a simple solution processed ultraviolet filtering layer, maintain 94% of their initial efficiency under simulated, 1 sun intensity, AM1.5 G irradiation for 1900 hours at 55 °C. Accelerated aging is also induced by exposure of light illumination intensities up to 27 suns, and operation temperatures as high as 65 °C. An extrapolated intrinsic lifetime of > 5.6 × 10^4^ h is obtained, which is equivalent to 30 years outdoor exposure.

## Abstract (web summary)

Through development of non-fullerene acceptors, OPVs have reached efficiencies of 18%, yet the inadequate operational lifetime still poses a challenge for the commercialisation. Here, the authors investigate the origin of instability of NFA solar cells, and propose some strategies to mitigate this issue.

## Introduction

Organic photovoltaic cells (OPVs) have sparked considerable interest in recent years owing to their lightweight, flexibility, low cost, and environmental friendliness. As distinguished from incumbent solar technologies, OPVs have shown promise for use in building-integrated energy generating windows and greenhouses due to their high absorption in the near infrared while being semitransparent, and importantly, neutral optical density in the visible^1–7^. Recently, ladder-type non-fullerene acceptors (NFAs) have led to OPV power conversion efficiencies (PCEs) of ~18% in opaque cells, and 10% in semitransparent cells with 50% visible transparency^8–13^. Although a few reports show that NFA-based solar cells under light-emitting diode (LED) illumination have the potential to reach long operational lifetimes (Supplementary Table 1), unfortunately, their ability to withstand use in harsh and realistic environments (significant ultraviolet (UV) infrared (IR), spectral content, and high operating temperatures) over long periods is, as yet, largely unproven^14–20^. In addition, high efficiency and long lifetime have yet to be simultaneously achieved in the same NFA-based cell. This has led to the belief that short operational lifetimes are an intrinsic disadvantage of high efficiency, NFA-based solar cells^14,15^.

Indeed, a pervasive myth associated with OPVs is that the materials are intrinsically vulnerable to degradation and morphological instabilities in the bulk heterojunction (BHJ) over the short term^21–23^. However, this myth is challenged by the very long extrapolated lifetimes (27,000 years) recently demonstrated in an archetype, thermally evaporated fullerene-based material system^24^, providing a proof-of-concept that OPVs can have exceptional operational lifetimes. Compared to the highly stable devices employing a C_70_ acceptor with its high bond-dissociation energy, the most efficient NFAs contain multiple thiophene units with relatively weak chemical bonds that are dissociated at high UV and IR light intensities^21–23^. Therefore, it remains an open question as to what the degradation mechanisms are of solution-processed systems based on NFAs. Beyond changes in the active BHJ in both materials and morphology, the properties of organic/electrode interfaces can also affect cell performance over time. Although strategies have been proposed to modify materials interfaces to suppress the degradation, to our knowledge, long term stable NFA-based solar cells under simulated air mass (AM) 1.5 G irradiation have not yet been realized^14–17^. The causes of instability for these high performance NFA systems are also still not well understood. This motivates and encourages us to investigate the correlation between materials, film morphology, device architecture, and their relationship to the reliability of NFA OPVs.

In this work, we study solution-processed, archetype single junction solar cells with efficiency competitive with similar acceptor-donor-acceptor (A-D-A) type NFA-based solar cells. Importantly, previously our group showed that semitransparent OPVs based on the same materials systems studied here can achieve high performance and potentially low cost when used in power generating windows^25,26^. This leaves the remaining unfulfilled promise of long operational lifetime. The solar cells show only a 6% loss in initial efficiency under simulated AM1.5 G, one sun intensity irradiation after 1900 h exposure. To facilitate data comparison between laboratories and, consequently, the identification of various degradation factors and failure mechanisms, ISOS level L-2 protocols are followed. These protocols are used to evaluate the reliability of NFA solar cells, which tests their intrinsic stability under illumination and at elevated temperatures^27^. Additionally, the aging is accelerated by exposure of the cells to illumination intensities as high as 27 suns, and temperatures up to 65 °C. The degradation rate is found to increase superlinearly with intensity, but there is no systematic dependence on temperature, leading to an extrapolated intrinsic lifetime, corresponding to a decrease in PCE of 20% from its initial value, of *T*_*80*_ > 5.6 ×10^4^ h, which is equivalent to 30 years of outdoor operation.

## Results

**Fig. 1:**
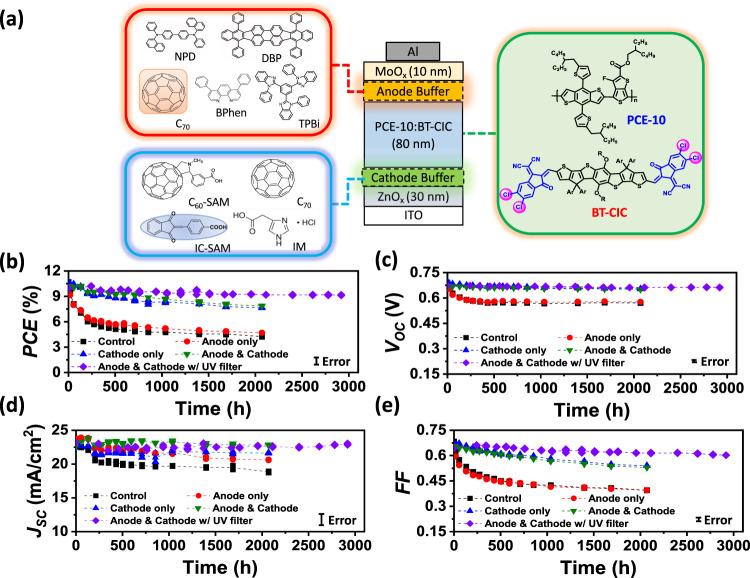
Device structure, molecular structures, and OPV ageing data under 1 sun simulated AM1.5 G illumination. **a** Schematic of the device showing layer thicknesses and compositions (right): molecular structural formulae of the PCE-10 and BT-CIC (left): molecular structural formulae of the cathode and anode buffer materials. **b** PCE power conversion efficiency, **c** *V*_*OC*_ Open-circuit voltage, **d** *J*_*SC*_ Short circuit current, and **e** *FF* Fill factor, plotted vs. aging time under 1 sun simulated AM1.5 G illumination for 3000 h with different device architectures (populations of 3–4 devices). The error bars indicate the 1 s.d. uncertainty of each measurement.

To investigate the reliability of NFA-based devices, we choose a material system used for semitransparent solar cell modules consisting of the commonly used donor, PCE-10, and an archetype near infrared (NIR) absorbing non-fullerene acceptor, BT-CIC, whose molecular structures are shown in Fig. 1a (see Methods for the nomenclatures of all molecules used in this study). An inverted device was fabricated with the structure: (ITO) / ZnO (30 nm) / PCE-10:BT-CIC (1:1.5, w/w, 80 nm) / MoO_x_ (10 nm) / Al (100 nm). To prevent chemical and morphological changes at organic/inorganic interfaces over time, buffer layers are inserted between the BHJ and charge transporting layers for improving the stability of the contact interface. As shown in Fig. 1a, several buffer materials are investigated for their influence on device reliability. The performance of the as-grown devices are listed in Supplementary Table 2 and 3. Figure 1b–e and Supplementary Fig. 1-2 shows the evolution of the packaged solar cell performance parameters over time under simulated AM1.5 G, one sun intensity illumination by Xe-arc lamp solar simulator at 55 ± 5 °C for both control and buffer-contained devices. All devices were encapsulated in a glovebox filled with ultrahigh purity nitrogen (<0.1 ppm O_2_ and H_2_O) by bonding a glass cover slide to the substrate using a bead of UV-curable epoxy around the substrate periphery (see Methods). Aging in a light soaking chamber was performed on the encapsulated devices in air at a relative humidity of ~50%.

As an example of the influence of buffer layers, a self-assembled monolayer (IC-SAM) and a 2 nm-thick C_70_ layer are applied at the electron and hole transporting layer interfaces with the BHJ, respectively. One group of cells was continuously exposed to one sun intensity, simulated AM1.5 G illumination at open circuit for 125 days, while the external quantum efficiencies (EQEs) and PCEs of the cells were periodically measured. A second, identical group of cells was aged at near the maximum power point (MPP). The control cell characteristics with no buffer layers decrease much faster, falling to less than 40% of their initial values within 2000 h, with *T*_*80*_ = 60 h, due to a rapid decrease in open-circuit voltage (*V*_*OC*_) and fill factor (*FF*). However, the decrease in short circuit current (*J*_*SC*_) is relatively minor, consistent with results reported for most NFA solar cells^16,27^. When the IC-SAM is deposited between the BHJ and the ZnO layer, the change in *V*_*OC*_ is suppressed, while *J*_*SC*_ and *FF* decrease slightly. The PCE retains 73% of its initial value after 2000 h. On the other hand, with the incorporation of an anode buffer (C_70_ or NPD) between BHJ and MoO_x_, the degradation of *J*_*SC*_ is reduced (Fig. 1d and Supplementary Fig. 2). The changes in *V*_*OC*_ and *FF*, however, remain similar to that of the control. Devices with both cathode and anode buffers exhibits significantly improved stability, with PCE reduced by <20% over 2000 h. Both *V*_*OC*_ and *J*_*SC*_ are stabilized with only a slight decrease in the *FF*. When a UV filter (with a wavelength cutoff at 400 nm) is attached on the distal surface of the substrate, the efficiency is further stabilized, remaining at 92% of its initial value after 3000 h. Notably, a similar result is observed between devices aged at open circuit and at MPP, as shown in Supplementary Fig. 3. The PCE retains 94% of its initial value after 2400 h. The changes in *J*_*SC*_ when measured under continuous illumination from a Xe-arc lamp are attributed to a redshift of the lamp spectrum with time^24^.

**Fig. 2:**
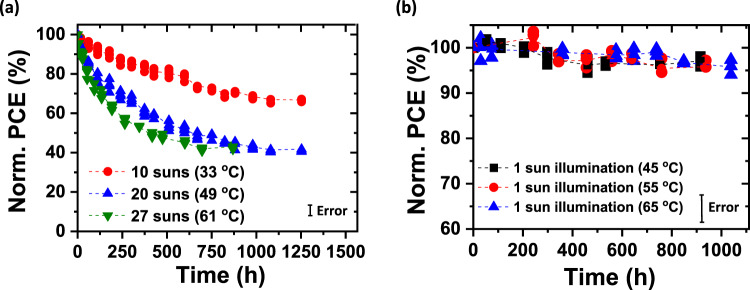
OPV efficiency under accelerated aging conditions. **a** Normalized PCE plotted vs. aging time under illumination equivalent to 10 ± 1.2, 20 ± 2.5, and 27 ± 3.8 suns. **b** Normalized PCE plotted vs. aging time under simulated AM1.5 G one sun illumination with temperature of 45 ± 5, 55 ± 5, and 65 ± 5 °C (populations of 3–4 devices). The error bars indicate the 1 s.d. uncertainty of PCE measurements.

To evaluate the efficacy of increasing temperature and light intensity in accurately predicting the stability of the OPVs under standard reporting conditions (1 sun intensity, AM1.5 G spectrum, 25 °C)^28^, the cells are exposed to light intensity to up to 27 suns using white light-emitting diodes (LEDs, see Methods). Provided that the effects of temperature and intensity on device aging are independent, the degradation acceleration factor given by^28^:

1 $$A={\left(\frac{{I}_{{{test}}}}{{I}_{{{ref}}}}\right)}^{\gamma }\exp \left[-\frac{{E}_{a}}{{k}_{B}}\left(\frac{1}{{T}_{{{test}}}}-\frac{1}{{T}_{a}}\right)\right]$$

allows for extrapolation to a reference intensity of *I*_*ref*_ = 1 sun when employing an elevated test intensity of *I*_*test*_, and for determining the lifetime at ambient temperature, *T*_*a*_, for a test done at *T*_*test*_. Here, *E*_*a*_ is the activation energy for failure, *k*_*B*_ is Boltzmann’s constant, and *γ* is the intensity-dependent acceleration factor. Figure 2a and Supplementary Fig. 4 show the performance characteristics of a population of OPV cells with both IC-SAM and C_70_ buffer layers aged at equivalent intensities of 10 ± 1.2, 20 ± 2.5, and 27 ± 3.8 suns. To avoid excessive heating at high intensity, the OPV cells were actively water-cooled to maintain the temperature at 33 ± 5, 49 ± 5, and 61 ± 5 °C, respectively. We observe that *J*_*SC*_ significantly decreases during aging, while *V*_*OC*_ and *FF* remain relatively stable. On the other hand, a second group of OPV cells with the same structure was aged by elevating the cell temperature under simulated AM1.5 G, one sun intensity illumination (see Methods). The evolution of solar cell performance over time is shown in Fig. 2b. We observed that the PCEs of the cells are stable, with no temperature-dependent increase in degradation rate up to 65 ± 5 °C.

While there are several sources for changes of OPV performance over time, none is more fundamental than the degradation of the organic molecules comprising the layer structure itself. To determine the chemical stability of the BHJ constituents, we performed nuclear magnetic resonance (NMR) spectroscopy on BT-CIC before and after aging under AM1.5 G one sun illumination (see Methods and Supplementary Fig. 5). There is no change after aging for 60 h, which suggests that the BT-CIC molecule itself is stable under light exposure. A similar stability was observed for PCE-10; its molecular weight remained unchanged and no fragments are observed in gel permeation chromatography spectra throughout the 50 h aging period (Supplementary Fig. 6). The photostability of PCE-10:BT-CIC blend film was investigated by UV-Vis spectroscopy (Supplementary Fig. 7). The shape and intensity of the exciton absorption spectrum shows almost no changes after aging for over 1500 h under AM1.5 G one sun illumination. This was also confirmed by Fourier-transform infrared spectroscopy as shown in Supplementary Fig. 8, where no difference was observed between the fresh and aged devices.

**Fig. 3:**
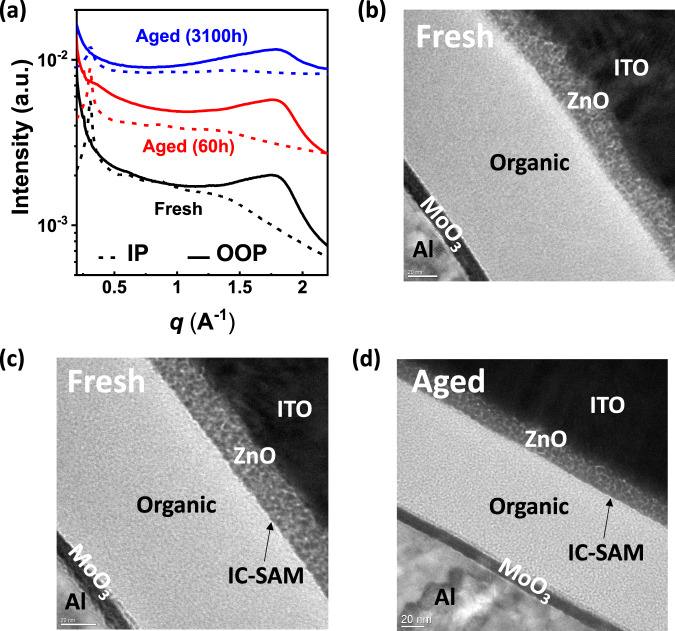
GIWAXS characterization and TEM images. **a** In-plane (dotted line) and out-of-plane (solid line) sector-averaged profiles extracted from grazing incidence wide-angle X-ray scattering (GIWAXS) patterns; *q* is the scattering vector. **b** Transmission electron microscope (TEM) image of cross-sectional slices of a fresh PCE-10:BT-CIC device without an interface buffer layer. **c** Fresh PCE-10:BT-CIC device with an IC-SAM layer inserted at the ZnO/BHJ interface. **d** Aged PCE-10:BT-CIC device with an IC-SAM layer inserted at the ZnO/BHJ interface under 27 ± 3.8 suns illumination for 870 h.

The morphological stability of the PCE-10:BT-CIC (1:1.5, w/w) film was further investigated using grazing incidence wide-angle X-ray scattering (GIWAXS), and carbon K-edge resonant soft X-ray scattering (R-SoXS) as functions of aging under AM1.5 G, 1 sun intensity illumination. As shown in Fig. 3a, the GIWAXS diffraction profile of the PCE-10:BT-CIC blend film shows a (010) peak in the out-of-plane direction at ~1.8 Å^−1^, and a (100) diffraction peak in the in-plane direction at 0.30 Å^−1^ characteristic of BT-CIC. No changes in coherence lengths were observed in the diffraction profiles after aging. Further, both fresh and aged PCE-10:BT-IC blends show the same multi-length-scale morphology with one peak at *q* = 0.10 nm^−1^ (corresponding to a distance of 63 nm) and another at 0.28 nm^−1^ (22 nm); see Supplementary Fig. 9, and a summary of the device performance over time in Table 1. Additionally, the composition of the BT-CIC molecule in the mixed domains was evaluated from the integrated scattering intensity (ISI)^29^, which scales with the standard deviation from the blend composition. This, in turn, is a function of the relative domain purity. The ISI remains constant over time, indicating that the bulk morphology of PCE-10:BT-CIC blend film is unchanged.

**Table 1:** Morphological parameters extracted from GIWAXS and R-SoXS measurements.

Sample	OPP (010) Peak	IP (100) Peak	q (nm^−1^)	Long period (nm)	ISI
Fresh	1.60/1.80	0.30	0.10	63	0.69
Aged (60 h)	1.60/1.80	0.30	0.10	63	0.70
Aged (3100 h)	1.59/1.80	0.30	–	–	–

Transmission electron microscopy (TEM) images of thin cross-sectional slices of the OPVs without, and with the IC-SAM interface buffer are shown in Fig. 3b–d, respectively. We find that the unprotected interface between ZnO and the BHJ appears broad, which is possibly due to the diffusion of ZnO into the organic layer. In contrast, when the IC-SAM is placed between the ZnO and the BHJ, we observe a sharp boundary between layers that is stable over time.

**Fig. 4:**
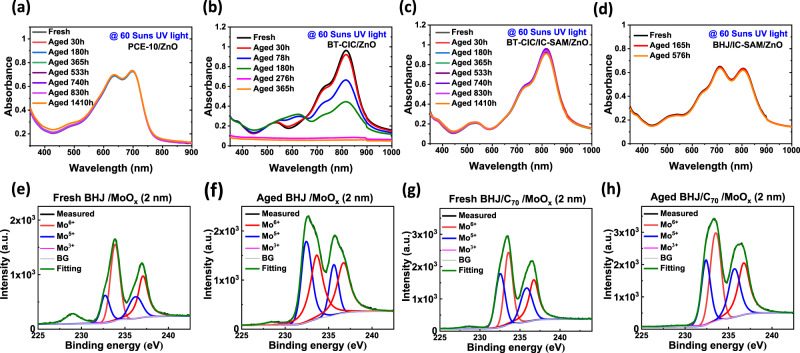
Assessment of the stability of the organic/inorganic interface. UV-Vis absorption spectra plotted vs. aging time under ultraviolet illumination with intensity equivalent to 60 suns of thin film of **a** PCE-10 on ZnO, **b** BT-CIC on ZnO, **c** BT-CIC on ZnO with an IC-SAM buffer, and **d** PCE-10:BT-CIC on ZnO with an IC-SAM buffer. X-ray photoelectron spectra of Mo *3d* for the fresh device without **e** 2 nm C_70_ layer, **f** aged device without C_70_ layer, **g** fresh device with C_70_ layer, and **h** aged device with C_70_ layer. The Gaussian distributions used to fit the spectrum and the sum of these Gaussians are shown by the solid lines. The black lines, which are often buried by the summation fit (green lines), correspond to the experimental results.

The absorption loss of encapsulated PCE-10 and BT-CIC on ZnO films aged under high-intensity UV (365 nm LED, 60 suns, equivalent) is shown Fig. 4a–d. Contrary to previous studies where PCE-10 on ZnO showed no degradation over 1400 h, UV soaking for ~30 h bleaches the BT-CIC. This is attributed to the photo-oxidation of the exocyclic double bond in BT-CIC that interrupts the π-conjugation, thus decreasing its absorbance^30,31^. With insertion of the IC-SAM, no bleaching of BT-CIC and PCE-10:BT-CIC blend films is observed, and the intramolecular charge transfer peak maintains both its shape and intensity (Fig. 4c, d and Supplementary Fig. 10). The X-ray photoelectron spectra (XPS) of the O *1* *s* core level are shown in Supplementary Fig. 11. We find the binding energy of oxygen at the ZnO/IC-SAM interface is blue-shifted compared to samples with only ZnO or IC-SAM, suggesting that stabilizing chemical bonds are formed between ZnO and IC-SAM. This was further verified by the C *1* *s* core XPS spectra, where an ester group is identified at the ZnO/IC-SAM interface (Supplementary Fig. 12). Such surface passivation results in the reduced photoluminescent (PL) intensity of an oxygen interstitial trap state in ZnO (Supplementary Fig. 13)^32^.

One common source of instability of organic materials is their dissociation when exposed to high energy (i.e., UV) radiation^22,33,34^. Therefore, we used matrix-assisted laser desorption ionization with time-of-flight mass spectrometry (MALDI-TOF-MS) to investigate photostability of BT-CIC by identifying the chemical fragments generated during the UV aging. The MALDI-TOF-MS analysis shows two new chemical species that exhibit lower mass in comparison to BT-CIC, see Supplementary Fig. 14. The peak at a mass-to-charge ratio, *m/z* = 1596.3, possibly originates from the scission of the C–O bond out of the thiophene backbone. The electron donating character of the oxygen atom is known to weaken the C–H bond, leading to the hydrogen abstraction on the CH_2_ group (α position to the oxygen)^22^. On the other hand, the peak at *m/z* = 1433.1 corresponds to a fragment with one peripheral phenyl group further removed. This result confirms that the weakest bonds in thiophene-based NFAs most susceptible to scission by UV irradiation are the side chain substituents.

## Discussion

There are three-primary sources of degradation of PCE-10:BT-CIC cells: photochemical reactions between the organic layers and the ZnO induced by exposure to UV illumination, increased concentration of defect states due to the penetration of Mo into the underlying organics (see Supplementary Fig. 15), and NFA bond dissociation when exposed to intense or prolonged UV radiation^35,36^. As discussed above, a monolayer of IC-SAM stabilizes the ZnO surface, thereby preventing photochemical reactions with the organics. Similarly, an ultrathin C_70_ layer inserted between the organics and MoO_x_ appears to substantially decrease anode-BHJ interactions. This is inferred from measurements of the Mo *3d* valence using X-ray photoelectron spectra (XPS) taken on PCE-10:BT-CIC (1:1.5, w/w, 80 nm)/MoO_x_ (2 nm) and PCE-10:BT-CIC (1:1.5, w/w, 80 nm)/C_70_ (2 nm)/MoO_x_ (2 nm) films before and after illumination for 250 h under simulated AM1.5 G, one sun intensity irradiation. As shown in Fig. 4e–h, the Mo *3d* spectra are fit by two *3d* doublets and a singlet, corresponding to Mo in three different oxidation states^36–38^. The peaks at 233.7 eV and 236.7 eV correspond to the *3d* doublet of Mo^6^^+^, those centered at 232.5 eV and 235.6 eV are the *3d* doublet of Mo^5^^+^, and the peak at 229.0 eV is attributed to Mo^3^^+^. It is apparent that the peak intensity of Mo^6^^+^ at the MoO_x_/BHJ interface decreases compared to the MoO_x_/2 nm C_70_/BHJ structure after light soaking. This suggests that the insertion of only 2 nm C_70_ between BHJ and MoO_x_ forms an effective barrier that prevents reduction of the Mo^6^^+^ to Mo^5^^+^, possibly by preventing the interdiffusion of thiophene-rich BT-CIC into MoO_x_. The conclusions drawn from these spectroscopic data are consistent with detailed analysis of the as-grown and aged *J–V* characteristics of OPVs under 1 sun, simulated AM1.5 G illumination both with, and without the peripheral buffer layers found in the Supplementary Fig. 16, where the photogeneration efficiency of the BHJ decreases continuously in the control device with no buffer layers. In contrast, the value remains almost constant in the buffered devices, suggesting that the stability of bulk morphology essential to a high device reliability is improved by employing buffer layers and a UV filter^39^.

To test if the methodology demonstrated in this work could be applied to the other NFA solar cells, we also fabricated devices based on other two archetype NFAs: BT-IC and Y6^25,40^. The evolution of solar cell performance over time under simulated AM1.5 G one sun illumination is plotted in Supplementary Figs. 17 and 18. Similar to the devices with BT-CIC, the decrease in *J*_*SC*_, *V*_*OC*_, and *FF* are suppressed in PCE-10:BT-IC when IC-SAM and C_70_ are inserted. Moreover, the degradation rate of OPV cells with buffer layers is at least 20 times less than in their absence without the elimination of UV effects. Additionally, the photostability of PM6:Y6 device was investigated^9^. The performance of device retains 90% of its initial value after aging for 500 h under AM1.5 G one sun illumination with a 400 nm long pass filter. This preliminary result indicates that NFA-based solar cells have the potential to meet the market needs of high reliability in addition to their high efficiency.

**Fig. 5:**
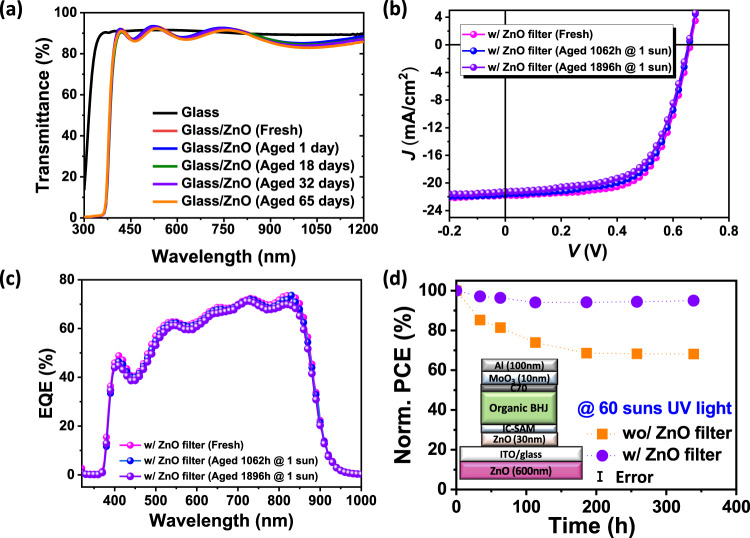
Characterization of the ZnO UV filter. **a** UV-Vis absorption spectra of a ZnO UV filter plotted vs. aging time under simulated AM1.5 G 1 sun illumination in air over 65 days. **b** Current-density-voltage characteristics, and **c** external quantum efficiency (EQE) spectra of fresh PCE-10:BT-CIC (1:1.5, w/w) devices with a ZnO UV filter, as well as the aged device under simulated AM1.5 G 1 sun illumination. **d** Normalized PCE plotted vs. aging time under ultraviolet illumination equivalent to 60 suns for more than 350 h with and without ZnO UV filter. The error bars indicate the 1 s.d. uncertainty of PCE measurements.

Since degradation depends on UV light exposure, we solution coat a 600 nm-thick ZnO film on the distal surface of the glass substrate that effectively blocks radiation at wavelengths <400 nm (Methods). Figure 5a shows the transmittance spectra of the fresh stand-alone filter, as well as one aged for 1500 h at 1 sun intensity in air. Compared to the bare glass substrate, the one comprising the ZnO filter exhibits negligible transmission at wavelengths <375 nm, with ~6% Fresnel reflection loss at >400 nm. As a result, an OPV with the ZnO filter is significantly more stable (Fig. 5b–d). The performance of the integrated device under 1 sun intensity AM1.5 G illumination retained 94% of its initial PCE after 1900 h Supplementary Fig. 19). Additionally, the integrated device is stable under extremely high UV powers (60 suns equivalent UV). The performance of the filtered device retained 95% of its initial PCE after 340 h, which is equivalent to 11 years of accumulated UV photons from outdoor exposure. In contrast, the PCE of the device lacking the UV filter declined by 66% over this same period.

**Fig. 6:**
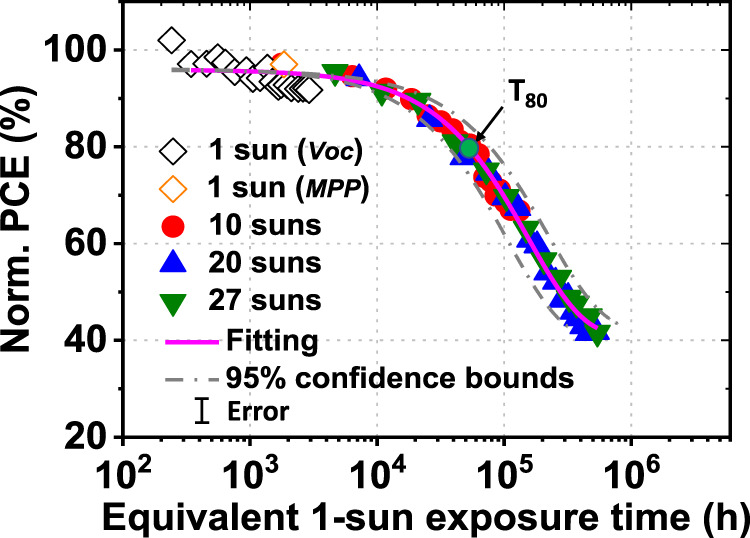
Aging acceleration factor and extrapolated OPV lifetime. Normalized PCE plotted vs. the equivalent 1 sun exposure time, defined as time (h) multiplied by intensity (suns) raised to the power of 2.02, for OPV cells under all illumination conditions used in this work. The data are fit to an exponential to estimate the time for the PCE to drop to 80% of its initial value, *T*_*80*_, with the best fit shown as a solid line. The error bar indicates the 1 s.d. uncertainty of the PCE measurements.

The UV-filtered devices with both C_70_ and IC-SAM buffer layers were then subjected to accelerated aging to determine their stability extrapolated to standard reporting conditions. As shown in Fig. 6, the normalized PCE is plotted vs. the equivalent 1 sun exposure time at intensities ranging from 1 to 27 suns. We find that results at all intensities follow the same trend described by Eq. 1 (shown by the fit line). Given the OPV cells are thermally stable up to at least 65 ± 5 °C, we can assume *E*_*a*_ ≈ 0 under reasonable ranges of temperature around the standard reporting conditions. Furthermore, from the fit to all data in Fig. 6, we obtain γ = 2.02 ± 0.16. We speculate that the quadradically dependent degradation of the PCE-10:BT-CIC BHJ cells results from second-order reactions such as exciton–exciton and exciton–polaron annihilation^41^. On the basis of this value of *γ*, we can define an equivalent 1 sun exposure time for OPV cells aged at each intensity. Extrapolating these data, we obtain a *T*_*80*_ > 5.6 × 10^4^ h. To convert the lifetimes of the OPV cells into outdoor lifetime projections, we assume an average of 5 kW-h/m^2^ of sunlight per day^42^. In this case, *T*_*80*_ extrapolates to >30 yr outdoor exposure, suggesting that the intrinsic stability of PCE-10:BT-CIC cells can substantially exceed the requirements for almost all practical applications.

This work demonstrates that NFA-based solar cells have the ability to achieve long term intrinsic stability under circumstances that use our innovative buffering and filtering schemes. However, to be practical, the cost and stability of encapsulation must also be considered. Although recent breakthroughs in deposition technology, such as atomic layer deposition (ALD), have led to dramatic improvements in the barrier to gas penetration, and hence improved reliability of organic light-emitting diodes (OLEDs), such layer growth is slow, and hence ultimately may be prohibitively costly^28^. Furthermore, barrier-coated plastic substrates are currently more costly to produce than metal foils or ultrathin glass substrates, and they also have compelling advantages such as toughness that may ultimately be more important than cost alone^28^. For transparent OPVs, however, the cost of encapsulation can be minimized by inserting the thin-film modules into the pocket of double-pane windows sealed with inert gas, as is common practice for insulating windows. The costs for such systems have been estimated to be quite attractive^26^.

In summary, we demonstrate a solution-processed non-fullerene, thiophene-based acceptor OPV with requirements of high efficiency, low cost, and remarkable operational stability simultaneously achieved. The instability of the NFA solar cells originates from time-dependent changes at the interfaces between the BHJ and the inorganic charge transporting layers. The changes are significantly suppressed by insertion of the ultrathin protective buffer layers, IC-SAM and C_70_, at each side of the BHJ. Further enhancement is afforded by the integration of a simple ZnO UV-filter layer to the distal surface of the glass substrate. This results in the PCE-10:BT-CIC solar cell that maintains 94% of its initial efficiency under simulated, 1 sun intensity, AM1.5 G irradiation for 1900 h at 55 °C. An extrapolated lifetime of *T*_*80*_ > 5.6 × 10^4^ h is obtained by exposure of light illumination intensities up to 27 suns, and operation temperatures as high as 65 °C. Our results show that NFA-based solar cells have the potential to meet the market needs of high reliability in addition to their high efficiency and potentially low manufacturing costs.

## Methods

### Materials

The non-fullerene acceptors are (4,4,10,10-tetrakis(4-hexylphenyl)-5,11-(2-ethylhexyloxy)-4,10-dihydro-dithienyl[1,2-b:4,5b′] benzodithiophene-2,8-diyl)bis(2-(3-oxo-2,3-dihydroinden-5,6-dichloro-1-ylidene)malononitrile, BT-CIC, and (4,4,10,10-tetrakis(4-hexylphenyl)-5,11-(2-ethylhexyloxy)-4,10-dihydro-dithienyl[1,2-b:4,5b′]benzodithiophene-2,8-diyl)bis(2-(3-oxo-2,3-dihydroinden-1-ylidene)malononitrile), BT-IC. The cathode buffer material is 4-((1,3-dioxo-1,3-dihydro-2H-inden-2-ylidene)methyl)benzoic acid, IC-SAM^25,38,43,44^. Other materials purchased from commercial suppliers are (4,4,10,10-tetrakis(4-hexylphenyl)-5,11-(2-ethylhexyloxy)-4,10-dihydro-dithienyl[1,2-b:4,5b′] benzodithiophene-2,8-diyl)bis(2-(3-oxo-2,3-dihydroinden-5,6-dichloro-1-ylidene) malononitrile, PCE-10, Poly[(2,6-(4,8-bis(5-(2-ethylhexyl-3-fluoro)thiophen-2-yl)-benzo[1,2-b:4,5-b’]dithiophene))-alt-(5,5-(1’,3’-di-2-thienyl-5’,7’-bis(2-ethylhexyl)benzo[1’,2’-c:4’,5’-c’]dithiophene-4,8-dione)], PM6, 2,2’-((2Z,2’Z)-((12,13-bis(2-ethylhexyl)-3,9-diundecyl-12,13-dihydro[1,2,5]thiadiazolo[3,4e]thieno[2”,3’‘:4’,5’]thieno [2’,3’: 4,5]pyrrolo[3,2-g]thieno[2’,3’:4,5]thieno[3,2-b]indole-2,10diyl)bis(methanylylidene))bis(5,6-difluoro-3-oxo-2,3-dihydro-1H-indene-2,1 diylidene))dimalononitrile, Y6 and 4-(1′,5′-Dihydro-1′-methyl-2′*H*-[5,6]fullereno-C_60_-I_h_-[1,9-c]pyrrol-2′-yl)benzoic acid, C_60_-SAM (1-Material, Dorval, CA), C_70_ SES Research, Houston, TX, US), 4-imidazoleacetic acid hydrochloride (Sigma–Aldrich, St. Louis, MO, US), N,N′-Di(1-naphthyl)-N,N′-diphenyl-(1,1′-biphenyl)-4,4′-diamine, NPD, 5,10,15,20-Tetraphenylbisbenz[5,6]indeno[1,2,3-cd:1′,2′,3′-lm]perylene, DBP, and 2,2’,2”-(1,3,5-Benzinetriyl)-tris(1-phenyl-1-H-benzimidazole), TPBi, Bathophenanthroline, Bphen from Luminescence Technology Corp., New Taipei City, Taiwan, MoO_3_ (Acros Organics, Fair Lawn, NJ, US), Zinc acetate dihydrate (Sigma–Aldrich, St. Louis, US) and Al (Alfa Aesar, Haverhill, MA, US).

### Solar cell fabrication

Prepatterned ITO-coated glass substrates with sheet resistances of 15 Ω/sq were purchased from Luminescence Technology Corp. Prior to thin-film deposition, the substrate surface was detergent and solvent cleaned with acetone and isopropanol, followed by CO_2_ snow cleaning and exposure to ultraviolet-ozone for 15 min^45^. The ZnO layer (ca. 30 nm) was spin cast from a ZnO precursor solution onto the substrates and then thermally annealed at 150 °C for 30 min in air. The IC-SAM was dissolved in methanol with a concentration of 1 mg/ml and spin coated at 4000 rpm for 60 s, followed by thermal annealing at 110 °C for 10 min. Then, the IC-SAM coated substrate was washed with methanol to remove the residues. The non-fullerene active layer, PCE-10:BT-CIC (1:1.5 by weight), was dissolved in chlorobenzene:chloroform (CB:CF, 10:1 by vol.) with a concentration of 16 mg/ml. The solution was filtered once with a 0.45 μm polytetrafluoroethylene (PTFE) syringe filter prior to use, and then spin-coated onto the substrate at 2000 rpm for 90 s to achieve a thickness of 80–90 nm. The samples were transferred into the vacuum chamber connected to glove boxes filled with ultrapure N_2_ (O_2_, H_2_O < 0.1 ppm). The C_70,_ MoO_3_ and Al films were deposited at 0.2 Å /s in a high vacuum chamber with a base pressure of 10^−7^ torr. The deposition rates and thicknesses were measured using quartz crystal monitors and calibrated post-growth by variable-angle spectroscopic ellipsometry. Device areas of 2 × 2 mm were defined by the overlap of the ITO anode and the Al cathode with an ultrathin shadow mask (50 µm).

The ZnO UV filter was deposited on the distal end of the glass substrate before the device fabrication. The ZnO layer (ca. 600 nm) was spun cast from a ZnO precursor solution and followed by thermal annealing at 300 °C for 1 h in air. After cooling to the room temperature, the ZnO-coated glasses were cleaned using a series of the solvents, acetone, and isopropanol, and sonicated 5 mins for each solvent.

### Solar cell characterization

The current–density–voltage (*J–V*) characteristics and external quantum efficiencies (EQE) of the cells were measured in a glovebox filled with ultrapure N_2_. The EQE measurements were performed with devices underfilled by a 200 Hz-chopped monochromated and focused beam from a Xe lamp. The current output from the devices as well as from a reference National Institute of Standards and Technology (NIST)-traceable Si detector were recorded using a lockin amplifier. Light from a Xe lamp filtered to achieve a simulated AM1.5 G spectrum (ASTM G173-03) was used as the source for *J–V* measurements. The lamp intensity is varied using neutral-density filters and was calibrated by a Si reference cell certified by National Renewable Energy Laboratory. Each cell was measured under six different light intensities from 0.001 sun to 1 sun (100 mW/cm^2^). The *J*_*SC*_ were calculated from the EQE spectrum, with <7% relative mismatch of the measured *J*_*SC*_ from *J–V* characteristics. The error bars quoted in the tables take into account both the random and systematic errors.

### Device stability measurements

All devices were encapsulated by bonding a glass cover slide to the substrate in a glovebox filled with high purity nitrogen, using an ultraviolet-curable epoxy. All aging in the light soaking chamber were performed on the encapsulated devices in air. The Xe-arc lamp spectrum (see Supplementary Fig. 20) was filtered to approximate an AM1.5 G reference, and its intensity was controlled to provide an output of 1.0 ± 0.1 kW m^−2^ during the aging period. One group of packaged devices was aged under open circuit at 55 ± 5 °C, and the photovoltaic efficiency was periodically recorded under AM1.5 G simulated spectrum. A second group of encapsulated devices was mounted on printed circuit boards and connected to a resistor to fix its current and voltage near the maximum power operating point. The PCE was recorded every 2 h. For the UV degradation mechanism studies, a 400 nm cutoff UV filter (Thorlabs) was placed between the OPV and the illumination source. Thermal stability tests were performed by aging the devices at open circuit between 45 ± 5 °C and 65 ± 5 °C in the light soaking chamber under 1 sun illumination. A resistive heater was placed on a Cu plate beneath each device to independently control temperature, which was monitored using a thermocouple. The photovoltaic efficiency was periodically recorded under AM1.5 G simulated spectrum.

High-intensity illumination from LEDs operating at various intensities was concentrated onto the OPVs with Ag-coated reflective light pipes. The LED intensity that produced a photocurrent equivalent to *J*_*SC*_ under AM1.5 G illumination (*J*_*SC*_, AM1.5 G) was equivalent to 1 sun intensity. To calibrate higher LED intensities, a neutral-density filter was used to ensure that the OPVs remained in their linear regime^24,46^. The neutral-density filter was 10% transmissive, thus the equivalent solar intensity (in units of suns) of the LEDs was calculated using the following equation:

2 $${{Equivalent}}\,{{solar}}\,{{intensity}}=\frac{10\,{J}_{{{photon,LED}}}}{{J}_{SC,{{AM1.5G}}}}$$

To avoid excessive heating at high intensity, the OPV cells were actively water-cooled with a closed-loop chiller to 33 ± 5, 49 ± 5, and 61 ± 5 °C at 10 ± 1.2, 20 ± 2.5, and 27 ± 3.8 suns, respectively. The ultraviolet-emitting LED intensity was measured using a calibrated Si photodiode and converted to a solar-equivalent intensity by dividing its output power by the power contained in the AM1.5 G spectrum at wavelengths <400 nm (4.6 W m^−2^). The responsivity of the Si photodiode was greater than 0.1 A W^−1^ over the wavelengths emitted by the ultraviolet LED. The spectra of the white LEDs and UV LED are shown in Supplementary Fig. 21.

### Molecular structure characterization

The ^1^H NMR spectra were collected using a Varian MR400 spectrometer in deuterated chloroform solution with trimethylsilane (TMS) as reference. Gel permeation chromatography (GPC) spectra were collected on a Shimadzu GPC system with THF as the solvent. The samples used for NMR and GPC measurements were fabricated on quartz and encapsulated with a cover glass similar to the OPV device. The package lids were removed after light soaking under 1 sun illumination. Laser desorption/ionization time-of-flight mass spectra were collected using a Bruker AutoFlex Speed MALDI-TOF instrument from the aged thin-film sample under nitrogen laser pulse illumination.

### Grazing incidence wide-angle X-ray scattering and resonant soft X-ray scattering measurements

Samples were prepared on Si wafers in a similar manner to the OPV devices. The thin films were measured at beamline 7.3.3 at the Advanced Light Source (ALS)^47^, Lawrence Berkeley National Lab (LBNL). The X-ray energy was 10 keV and the scattering patterns were recorded on a 2D image plate (Pilatus 1 M) with a pixel size of 172 μm (981 × 1043 pixels). The samples were ~10 mm long in the direction of the beam path, and the detector was located at a distance of 300 mm from the sample center (distance calibrated by a AgB reference). The incidence angle was 0.16°. Resonant soft X-ray scattering with photon energy of 283.6 eV was performed at beamline 11.0.1.2 of LBNL^48^. Thin films were transferred onto a Si_3_N_4_ substrate and the experiment was done in the transition mode.

### Transmission electron microscopy (TEM)

The cross-sectional slice of the OPV device was prepared by focused ion beam (FIB) milling (FEI Nova 200 Nanolab SEM/FIB) and glued by Pt soldering onto a FIB lift-out grid rod. The sample was then transferred into a TEM chamber (JEOL 2010F) to take images.

### Thin-film absorption and transmission measurements

The absorbance and transmittance of thin films were collected using a calibrated PerkinElmer Lambda 1050 ultraviolet-visible spectrometer.

### X-ray photoelectron measurements (XPS)

XPS spectra were collected using a Kratos Axis Ultra XPS at the Michigan Center for Materials Characterization, with a monochromatic Al source (10 mA, 14 kV), a pass energy of 20 eV and step size of 0.1 eV on a spot size of 700 × 300 µm. The XPS curves were analyzed with CasaXPS software. To account for charge compensation, the C *1* *s* peak at 284.5 eV was used to calibrate the energies. Each peak was analyzed using Gaussian-Lorentzian curves with a full-width half maxima less than two and a linear background.

### Photoluminescence measurements

The PL spectra were measured using a 325 nm He-Cd continuous wave laser with a pump power ~ 50 µW. A notch filter at 325 ± 5 nm was used to filter the background emission from the laser, and an additional 400 nm long pass filter is used to filter the pump laser at the end of the signal path. The output signal was coupled to the CCD via an optical fiber and monochromator. All samples used for PL measurement were prepared on the quartz substrates.

## Supplementary information

Supplementary Information

## Data Availability

The data supporting the results of this work are available from the corresponding authors upon reasonable request.
