# Supplementary material for: Non-fullerene acceptor organic photovoltaics with intrinsic operational lifetimes over 30 years
Source: Nat Commun. 2021 Sep 14;12:5419. doi: 10.1038/s41467-021-25718-w (PMC8440764; doi:10.1038/s41467-021-25718-w)
Supplement: Supplementary file 1 — Supplementary Information [file 41467_2021_25718_MOESM1_ESM.pdf]

**Supplementary Information**

# Non-fullerene Acceptor Organic Photovoltaics with Intrinsic Operational Lifetimes over 30 Years

Yongxi Li[1], Xiaheng Huang[1], Kan Ding[1], Hafiz K. M. Sheriff, Jr. [2], Long Ye[3,4], Haoran Liu[5], Chang-Zhi Li[5], Harald Ade[3] and Stephen R. Forrest[1,2*]

[1]*Departments of Electrical Engineering, Material Science and Engineering, and Physics, University of Michigan, Ann Arbor, Michigan 48109, USA*

[2]*Applied Physics Program, University of Michigan, Ann Arbor, Michigan 48109, USA*

[3]*Department of Physics and Organic and Carbon Electronics Laboratories (ORaCEL), North Carolina State University, Raleigh, NC 27695, USA*

[4]*School of Materials Science and Engineering and Tianjin Key Laboratory of Molecular Optoelectronic Sciences, Tianjin University, Tianjin 300072, China*

[5]*State Key Laboratory of Silicon Materials, MOE Key Laboratory of Macromolecular Synthesis and Functionalization, Department of Polymer Science and Engineering, Zhejiang University, Hangzhou, 310027, China*

**Supplementary Table 1.** Comparison of the stability of NFA OPVs in our work with some recent reports. $PCE_0$ and PCE(t) are the efficiency of devices before and after stability tests.

| Device Structure | Light Source | Aging condition | Degradation factor | $PCE_0$ (%) | PCE(t)/ $PCE_0$ | Estimated $T_{80}$ (h) | Ref |
|---|---|---|---|---|---|---|---|
| ZnO/ITO/ZnO/IC-SAM/PCE-10:BT-CIC/$C_{70}$/MoOx/Al | Xe Arc (200-2800 nm) | Encap, 1900 h open-circuit, 55℃ | Light & Thermal | 9.8 | 94% | 56,000 | This work |
| ITO/ZnO/IC-SAM/PCE-10:BT-CIC/$C_{70}$/MoOx/Al | Xe Arc w/ filter (400-2800 nm) | Encap, 2400 h MPP, 55℃ | Light & Thermal (wo/UV) | 10.2 | 94% | 56,000 | This work |
| ITO/ZnO/IC-SAM/PCE-10:BT-CIC/$C_{70}$/MoOx/Al | Xe Arc w/ filter (400-2800 nm) | Encap, 3000 h open-circuit, 45, 55 & 65℃ | Light & Thermal (wo/UV) | 10.2 | 92% | 56,000 | This work |
| ITO)/ZnO/P3HT:IDTBR/ PEDOT:PSS/Ag | White LED (410-780 nm) | $N_2$, 2000 h open-circuit | Light (wo/UV & IR) | 6.1 | 95% | N/A | (1) |
| ITO/ZnO/ PBDB-T:ITIC-2F /MoOx/Al | White LED (410-780 nm) | $N_2$, 1600 h open-circuit, < 40℃ | Light (wo/UV & IR) | 7.8 | 85% | 11,000 | (2) |
| ITO/ZnO/ PM6:IT-4F /MoOx/Al | White LED (410-780 nm) | $N_2$, 1600 h open-circuit, < 40℃ | Light (wo/UV & IR) | 10.8 | 76% | 1300 | (3) |
| ITO/ZnO/ P3HT:*o*-IDTBR/MoOx/Al | Metal halide (380-800 nm) | $N_2$, 2000 h open-circuit, 25℃ | Light (wo/UV & IR) | 5.0 | 92% | N/A | (4) |
| ITO/ZnO/ C60-SAM/ PTB-7-Th:IEICO-4F/MoOx/Al | LED (350-980 nm) | Encap, 2000 h open-circuit, 25℃ | Light (wo/UVB & IR) | 9.9 | 105% | 34,000 | (5) |
| ITO/ZnO/SAM/PBDBT:IT-IC/MoOx/Ag | Metal halide (330-780) | Encap, 180 h open-circuit, 25℃ | Light (wo/UVB & IR) | 10.1 | 70% | N/A | (6) |
| ITO/PEDOT:PSS/ PM6:PM7:Y6:$PC_{71}BM$/PFNDI-Br/Ag | LED (N/A) | N/A, 1000 h | Light | 17.7 | 81% | N/A | (7) |
| ITO/ZnO/NDI-B/PBDB-TF:BTP-eC9/MoOx/Al | White LED (410-780 nm) | Encap, 1800 h open-circuit, 50℃ | Light (wo/UV & IR) | 17.0 | 93% | N/A | (8) |
| ITO/2PACz/ PM6:N3/PFNBr/Ag | White LED (410-780 nm) | Vacuum, 120 h MPP | Light (wo/UV & IR) | 16.3 | 74% | N/A | (9) |
| ITO/ZnO/PM-6:IT-4F/MoOx/Ag | Xe Arc (200-2800 nm) | $N_2$, 24 h open-circuit | Light | 13.5 | 89% | N/A | (10) |
| ITO/ZnO/PEI/PM6:Y6:$PC_{71}BM$/MoOx/Ag | Xe Arc (200-2800 nm) | $N_2$, 1000 h open-circuit | Light | 15.4 | 75% | N/A | (11) |
| ITO/ZnO/Py-BDP/PM-6:IT-4F/MoOx/Al | Xe Arc (200-2800 nm) | Encap, 9 h open-circuit, 25℃ | Light | 11.2 | 66% | N/A | (12) |
| ITO/ZnO/PBDBT:IT-IC/MoOx/Ag | Xe Arc (200-2800 nm) | Encap, 1100 h open-circuit, 25℃ | Light | 10.1 | 25% | N/A | (13) |

**Supplementary Table 2.** Operating characteristics of OPVs based on PCE-10:BT-CIC (1:1.5, w/w) with different anode buffer layers under simulated of AM 1.5G, 100 mW/cm$^2$, illumination.

| Buffers | $J_{sc}$ [mA/cm$^2$] | $V_{OC}$ [V] | *FF* [%] | *PCE* [%] |
|---|---|---|---|---|
| $C_{60}$-SAM:$C_{70}$ | 22.40±0.11 | 0.663±0.007 | 64.5±0.4 | 9.59±0.12 |
| $C_{60}$-SAM:DBP | 22.70±0.09 | 0.669±0.003 | 63.3±0.2 | 9.61±0.05 |
| $C_{60}$-SAM:NPD | 23.96±0.21 | 0.678±0.003 | 64.4±0.3 | 10.46±0.13 |
| $C_{60}$-SAM:Bphen | 21.52±0.01 | 0.312±0.01 | 43.4±0.3 | 2.90±0.19 |
| $C_{60}$-SAM: TPBi | 23.46±0.25 | 0.481±0.01 | 53.4±0.2 | 6.01±0.19 |

**Supplementary Table 3.** Operating characteristics of OPVs based on PCE-10:BT-CIC (1:1.5, w/w) with different cathode buffer layers under simulated of AM 1.5G, 100 mW/cm$^2$, illumination.

| Buffers | $J_{sc}$ [mA/cm$^2$] | $V_{OC}$ [V] | *FF* [%] | *PCE* [%] |
|---|---|---|---|---|
| IC-SAM:$C_{70}$ | 23.25±0.14 | 0.672±0.001 | 65.7±0.4 | 10.2±0.09 |
| $C_{70}$:$C_{70}$ | 23.81±0.24 | 0.652±0.002 | 63.4±0.2 | 9.80±0.10 |
| IM :$C_{70}$ | 16.58±0.07 | 0.673±0.001 | 62.3±0.1 | 6.95±0.07 |
| C60-SAM:$C_{70}$ | 22.40±0.11 | 0.663±0.002 | 64.5±0.4 | 9.59±0.12 |

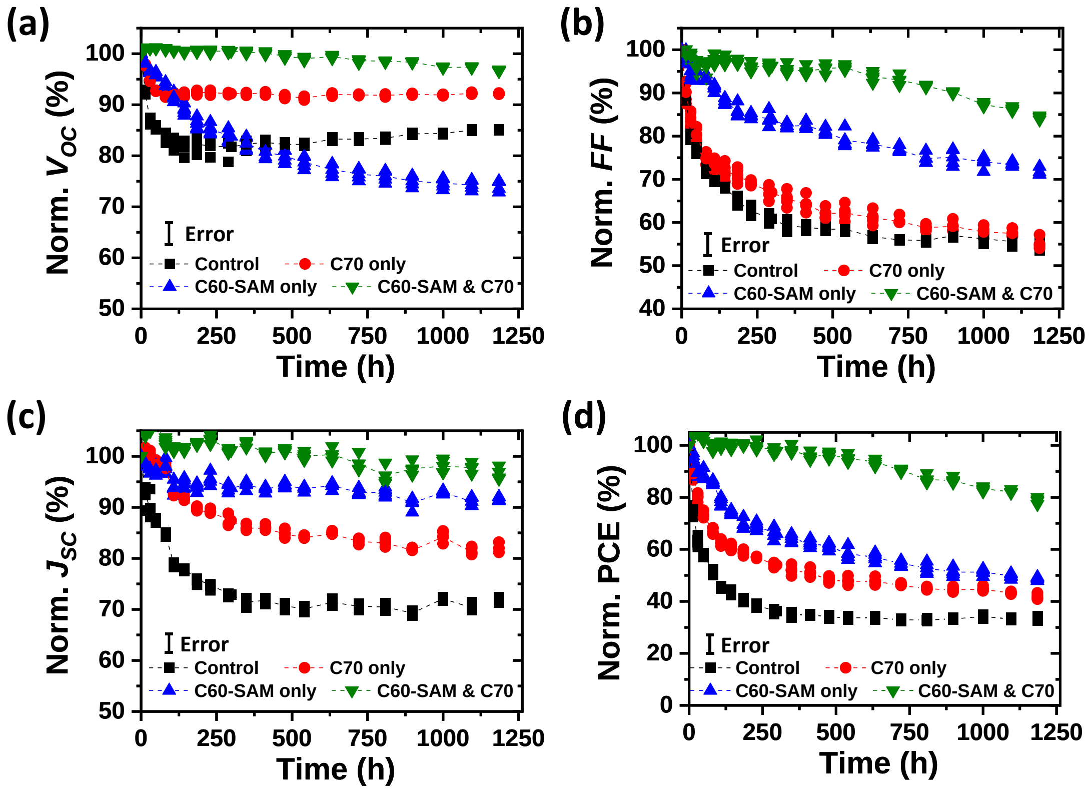


**Supplementary Figure 1.** Normalized (a) $V_{OC}$, (b) $FF$, (c) $J_{SC}$ and (d) PCE of a PCE-10:BT-CIC (1:1.5, w/w) OPV plotted vs aging time under 1-sun simulated AM1.5G illumination with $C_{60}$-SAM as a cathode buffer and $C_{70}$ as an anode buffer (averaged for 4 devices). The error bars indicate the 1 s.d. uncertainty of each measurement.

.

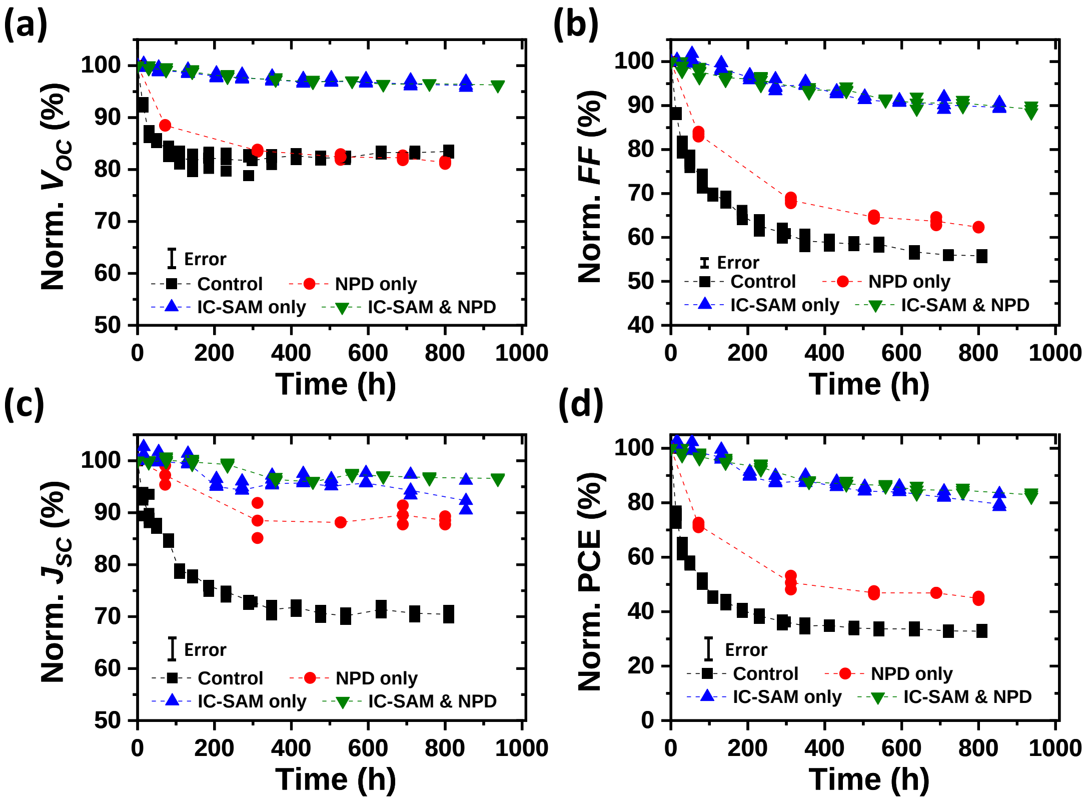


**Supplementary Figure 2.** Normalized (a) $V_{OC}$, (b) *FF*, (c) $J_{SC}$ and (d) PCE of a PCE-10:BT-CIC (1:1.5, w/w) OPV plotted vs. aging time under 1-sun simulated AM1.5G illumination with IC-SAM as a cathode buffer and NPD as an anode buffer (averaged for 3-4 devices). The error bars indicate the 1 s.d. uncertainty of each measurement.

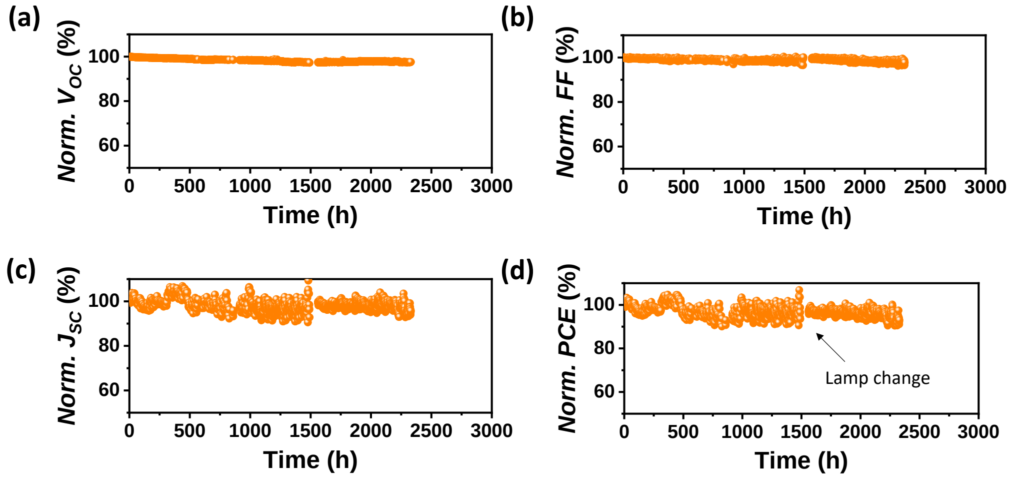


**Supplementary Figure 3.** Normalized (a) $V_{OC}$, (b) *FF*, (c) $J_{SC}$ and (d) PCE of an PCE-10:BT-CIC (1:1.5, w/w) vs. against aging time at near the maximum power point under 1-sun simulated AM1.5G illumination with IC-SAM as a cathode buffer, $C_{70}$ as an anode buffer and a 400 nm cutoff UV-filter.

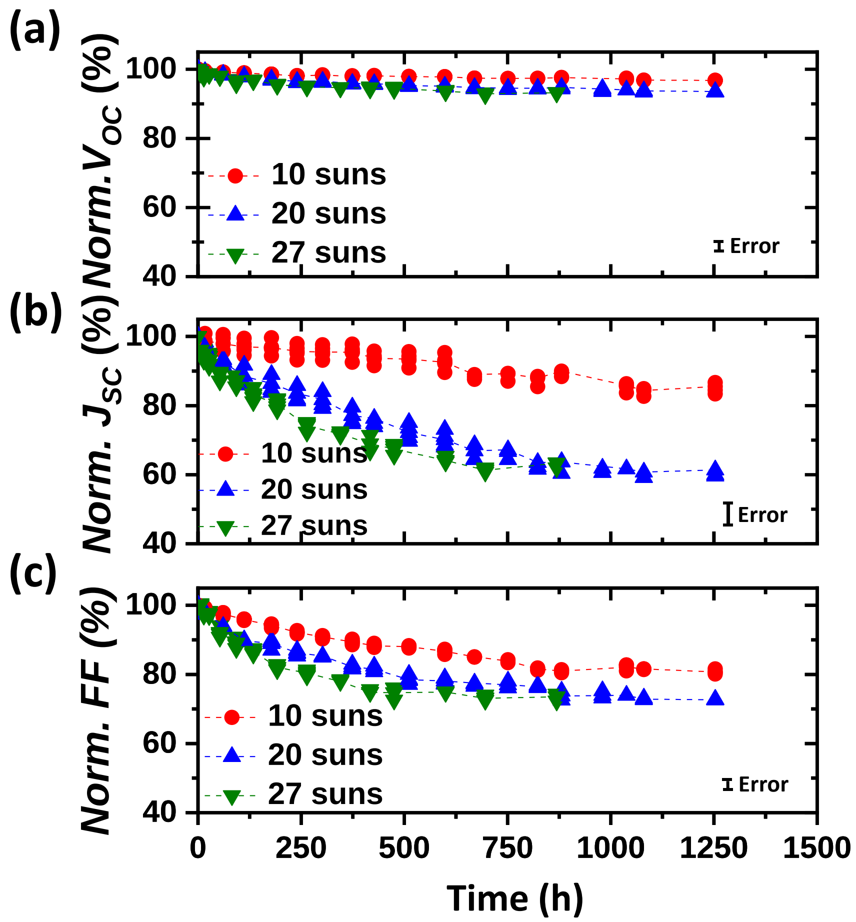


**Supplementary Figure 4.** Normalized (a) $V_{OC}$, (b) $J_{SC}$ and (c) *FF* of a PCE-10:BT-CIC (1:1.5, w/w) OPV with IC-SAM as a cathode buffer, $C_{70}$ as an anode buffer plotted vs. aging time under illumination equivalent to 10 ± 1.2, 20 ± 2.5 and 27 ± 3.8 suns (averaged for populations of 2-4 devices). The error bars indicate the 1 s.d. uncertainty of each measurement.

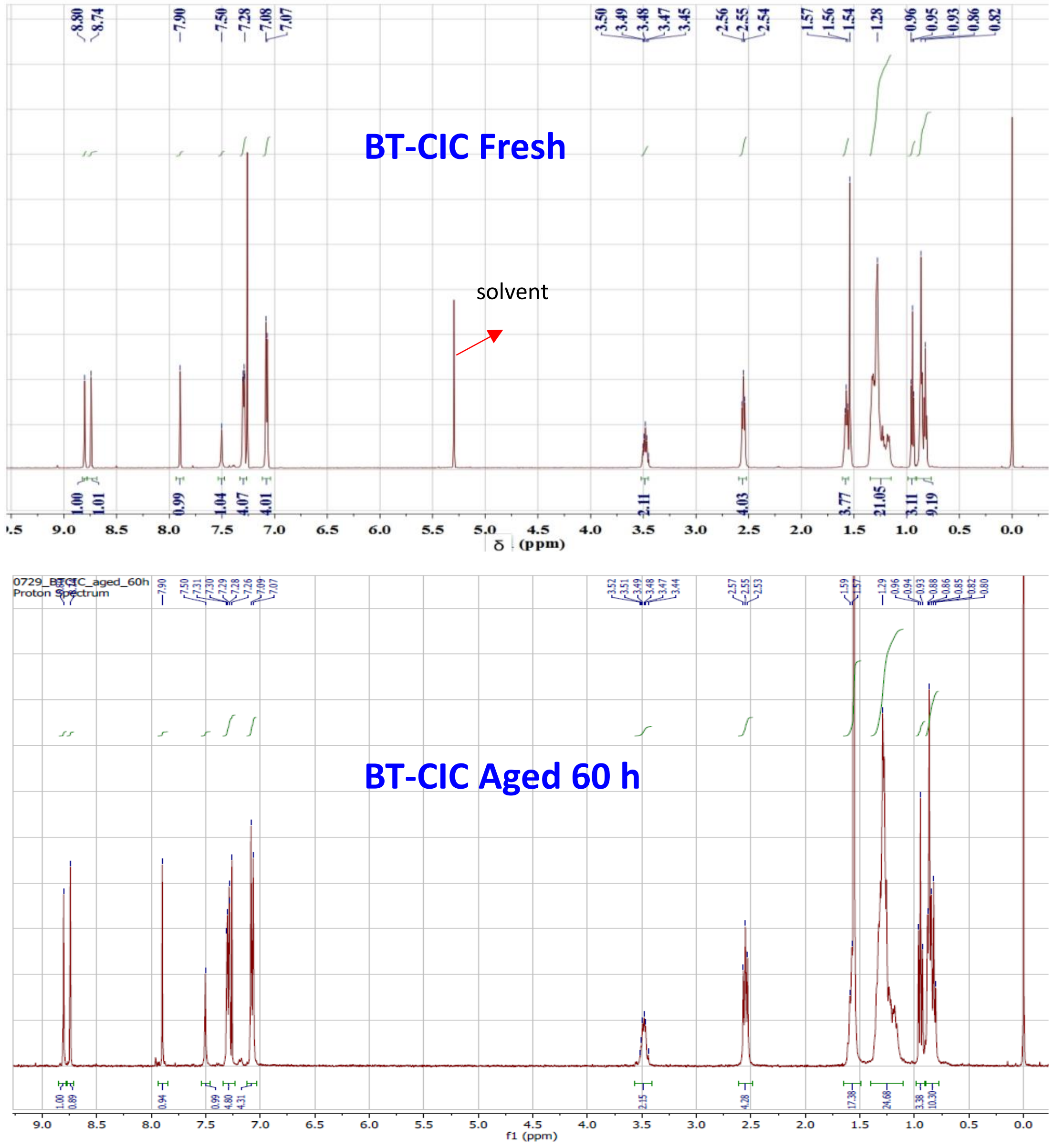


**Supplementary Figure 5.** $^{1}H$ NMR spectra of BT-CIC fresh and aged samples in $CDCl_3$.

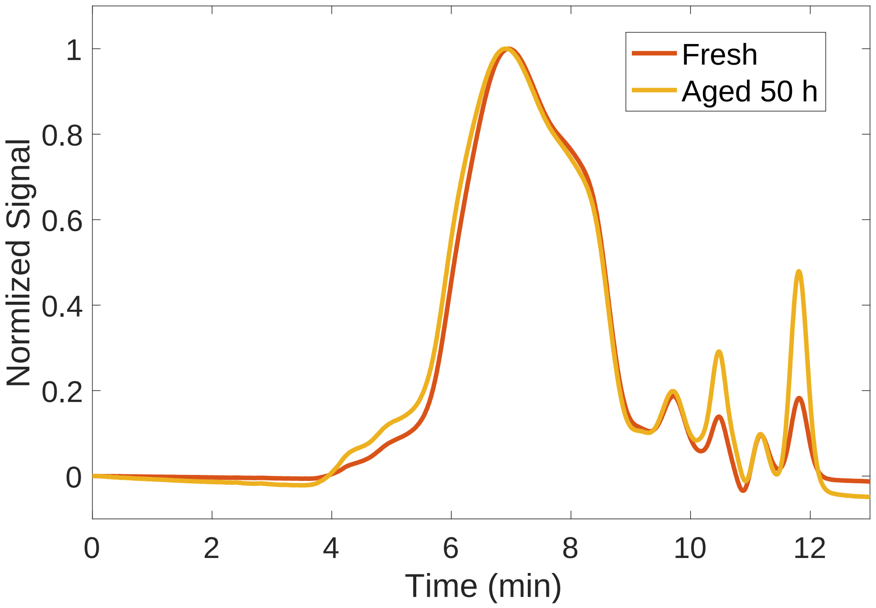


**Supplementary Figure 6.** Molecular weight spectra of PCE-10 fresh and aged samples using gel permeation chromatography with THF as solvent.

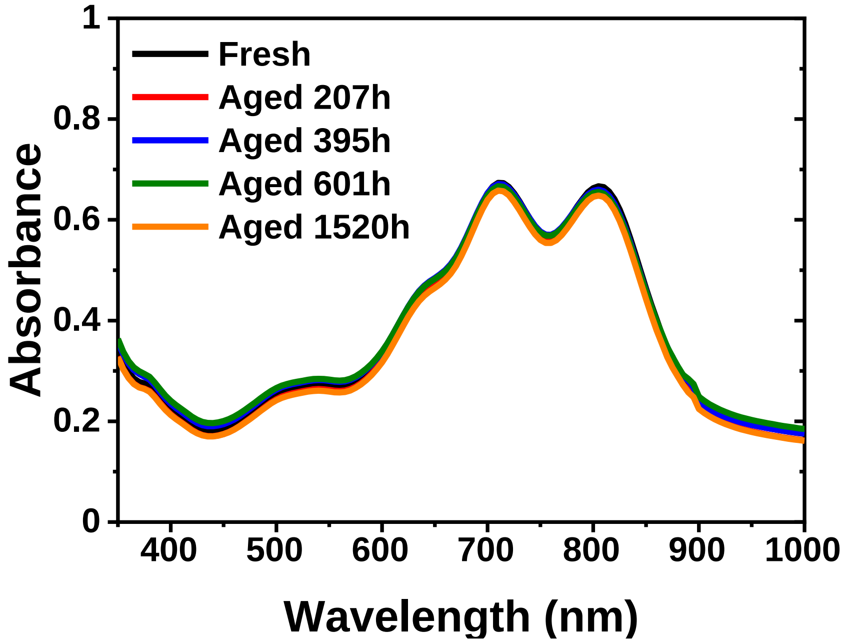


**Supplementary Figure 7.** UV-Vis absorption spectra plotted vs. aging time of an encapsulated PCE-10:BT-CIC (1:1.5, w/w) thin film on the quartz under 1-sun illumination.

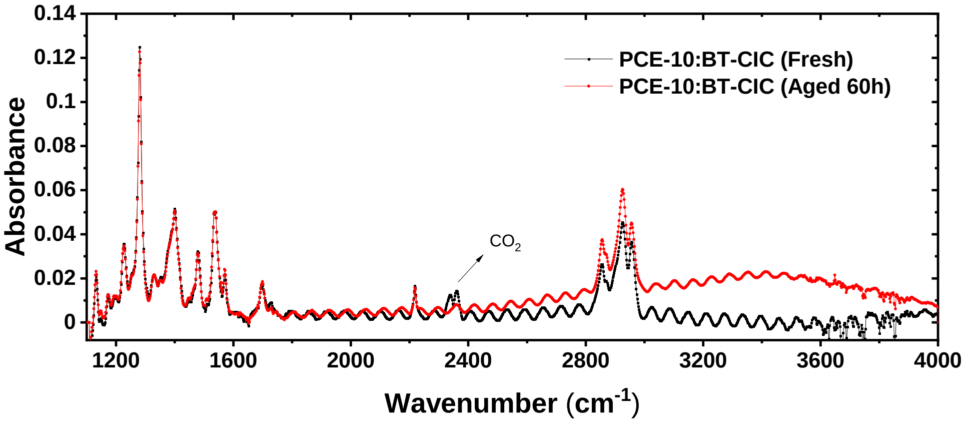


**Supplementary Figure 8.** Fourier-transform infrared spectroscopy (FTIR) plotted vs. aging time of an encapsulated PCE-10:BT-CIC (1:1.5, w/w) thin film on the $CaF_2$ under 1-sun illumination.

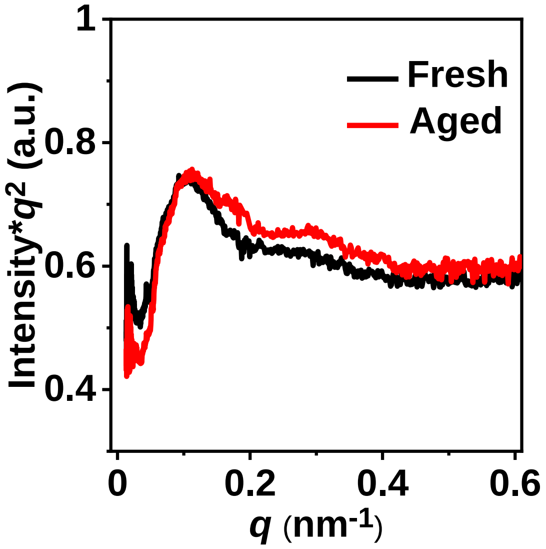


**Supplementary Figure 9.** Lorentz corrected and thickness normalized resonant soft x-ray scattering profiles of fresh and aged PCE-10:BT-CIC blend under simulated AM1.5G 1-sun illumination for 60 h. Here, $q$ is the scattering vector.

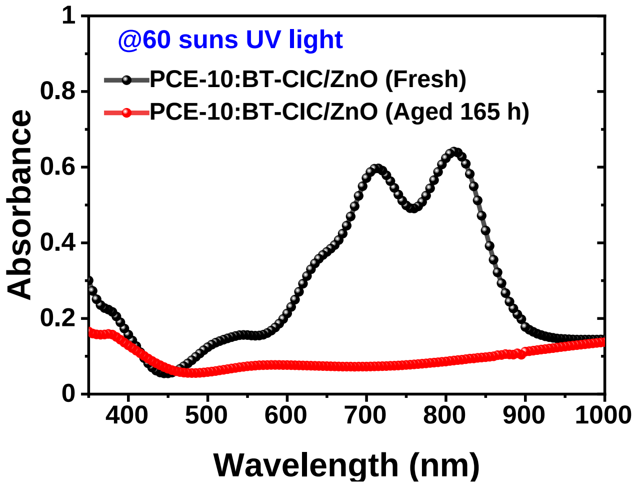


**Supplementary Figure 10.** UV-Vis absorption spectra plotted vs. aging time of an encapsulated PCE-10:BT-CIC (1:1.5, w/w) thin film on the ZnO under 60-sun UV illumination.

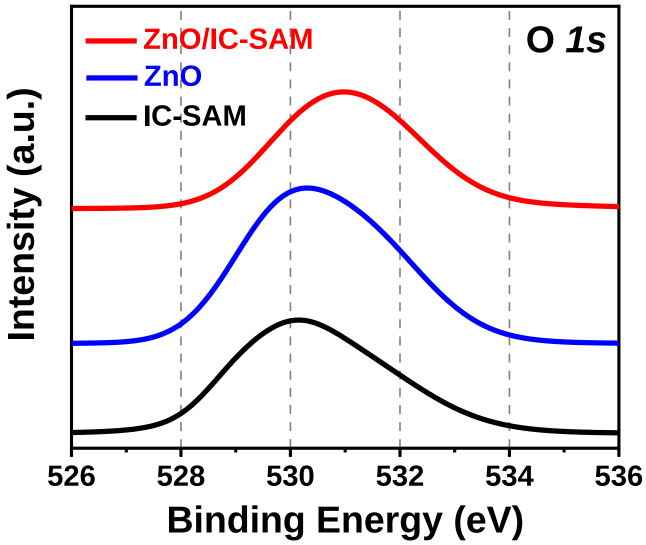


**Supplementary Figure 11.** The X-ray photoelectron spectra of O *1s* for the ZnO/IC-SAM, ZnO and IC-SAM films deposited on ITO glass.

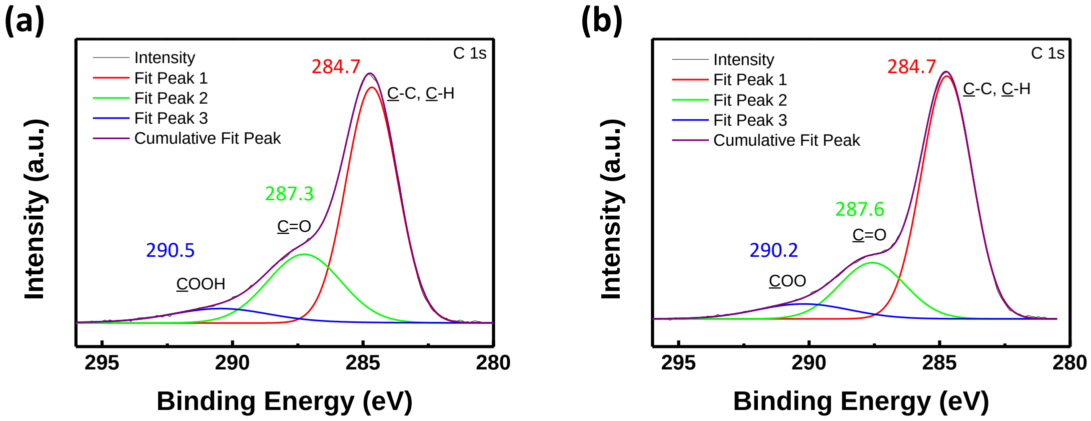


**Supplementary Figure 12.** The X-ray photoelectron spectra of C *1s* for the (a) IC-SAM, and (b) ZnO/IC-SAM films deposited on ITO glass.

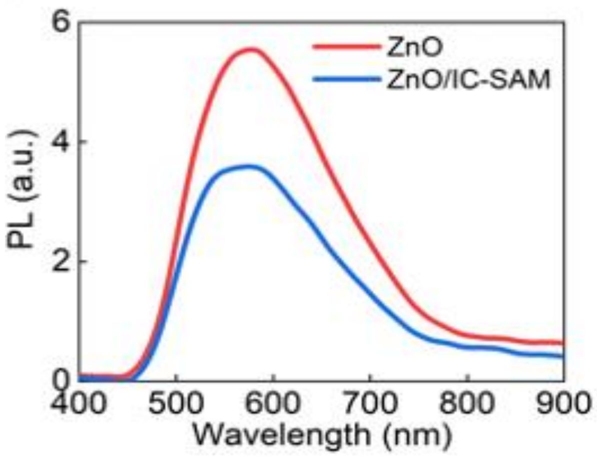


**Supplementary Figure 13.** Photoluminescence spectra of ZnO and ZnO/IC-SAM films deposited on quartz with an additional 400 nm long pass filter.

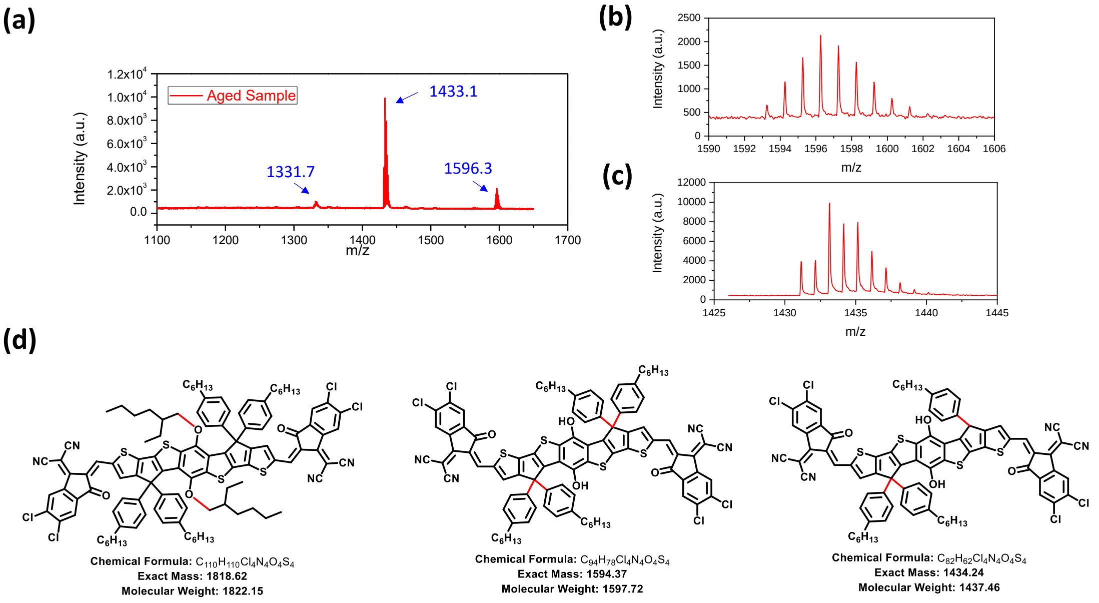


**Supplementary Figure 14.** (a) MALDI-TOF mass spectrometry of aged BT-CIC, (b) Detail of molecular fragments with m/z= 1596.3, and (c) m/z= 1433.1, (d) Molecular products proposed for BT-CIC is dissociated into the fragment with m/z= 1596.3, illustrated in red, and then further dissociated into m/z=1433.1. The calculated molecular weight is shown in below each molecular structure.

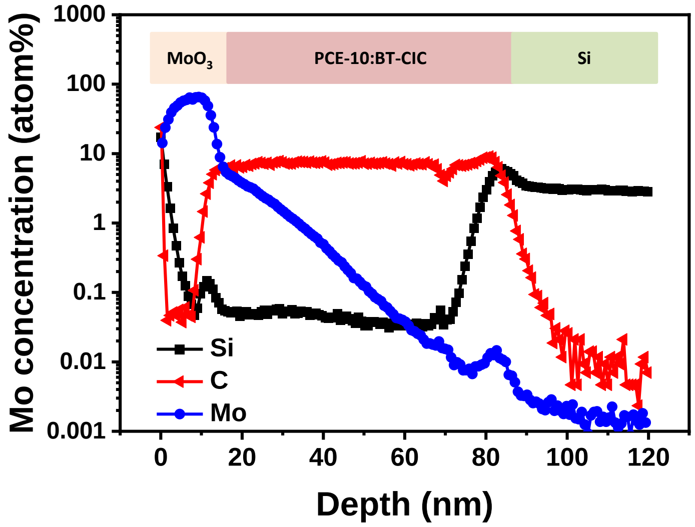


**Supplementary Figure 15.** Time of flight secondary-ion mass spectra of a device comprising a PCE-10:BT-CIC bottom layer and $MoO_3$ top layer.

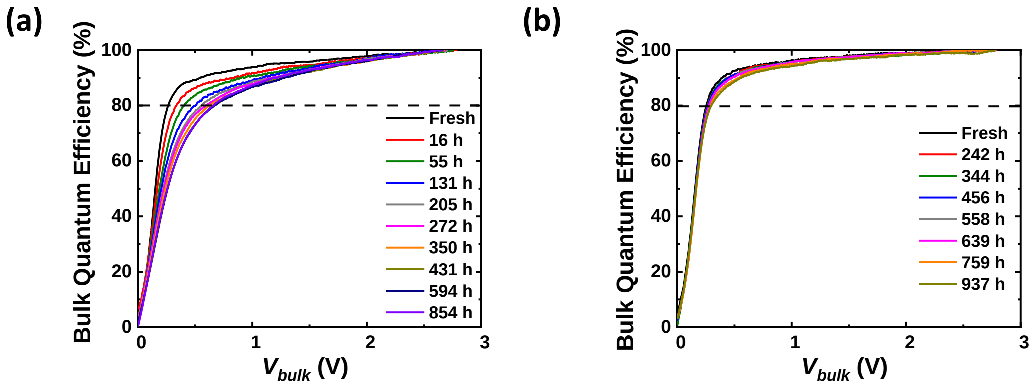


**Supplementary Figure 16.** Bulk quantum efficiency - $V_{bulk}$ characteristics of the aged devices with identical BHJs comprising PCE-10 as the donor and BT-CIC as the acceptor (1:1.5, w/w, 80 nm) and (a) control device, (b) the device with IC-SAM as a cathode buffer, $C_{70}$ as an anode buffer and a 400 nm cutoff UV-filter.

**Supplementary Note 1.** The stability of the bulk morphology is also evident from the calculation of the photogeneration efficiency of the BHJ, known as the bulk quantum efficiency (BQE); a quantity that only measures the photogeneration properties of the BHJ. Specifically, the BQE and does not depend on contributions to the photo response from the layers peripheral to the BHJ (i.e. the electrodes, buffer layers and interfaces). The BQE is given by[14]:

$$BQE\ (V_{bulk}) = \frac{J_{ph}(V_{off} - V_{bulk})}{J_{sat}}$$

where $V_{bulk}$ is the voltage across the BHJ, $J_{\mathrm{ph}}$ is the photocurrent, $V_{off}$ is the applied voltage at which $J_{ph}$ is zero, $J_{sat}$ is the saturated photocurrent at a large reverse bias. In this work, $J_{sat}$ is determined at −2 V. For ease in comparing the BQE– $V_{bulk}$ data, $V_{80}$ is defined as $V_{bulk}$ required to achieve 80% of the maximum cell photogeneration efficiency measured in the 4$^{th}$ quadrant of the J-V characteristics under 1 sun, AM1.5G simulated illumination. The smaller the $V_{80}$ is, the more easily the photogenerated charges are extracted from the BHJ.

To evaluate the efficacy of the buffering and filtering schemes, we fabricated both control and buffered devices with the following respective structures: ITO / ZnO (30 nm) / PCE-10:BT-CIC (1:1.5, w/w, 80 nm) / $MoO_x$ (10 nm) / Al (100 nm) and ITO / ZnO (30 nm) / IC-SAM / PCE-10:BT-CIC (1:1.5, w/w, 80 nm) / $C_{70}$ (2 nm) / $MoO_x$ (10 nm) / Al (100 nm). **Figure S16** shows the time evolution of the BQE-$V_{bulk}$ characteristics of both devices aged for approximately 900 h under AM 1.5G one sun illumination. The BQE decreases continuously in the control device, while $V_{80}$ increases from 0.26 V to 0.67 V. In contrast, $V_{80}$ remains almost constant for the devices with

buffer and UV filter layers, suggesting that there is no significant morphology change that occurs in the BHJ.

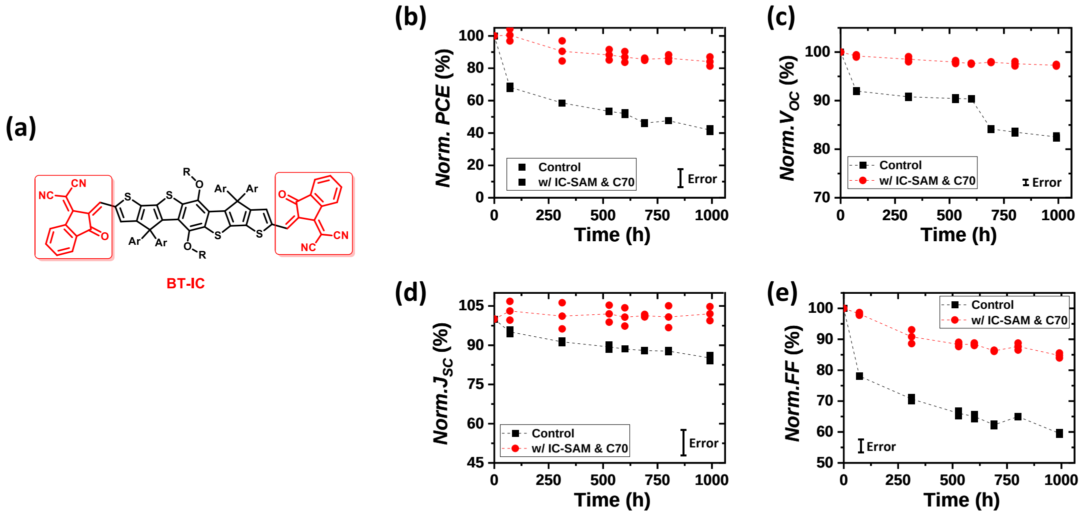


**Supplementary Figure 17.** (a) Molecular structural formulae of BT-IC, (b) Normalized PCE, (c) $V_{OC}$, (d) $J_{SC}$ and (e) *FF* of a PCE-10:BT-IC (1:1.5, w/w) OPV plotted vs. aging time under 1-sun simulated AM1.5G illumination with IC-SAM as a cathode buffer and $C_{70}$ as an anode buffer (averaged for 4 devices). The error bars indicate the 1 s.d. uncertainty of each measurement.

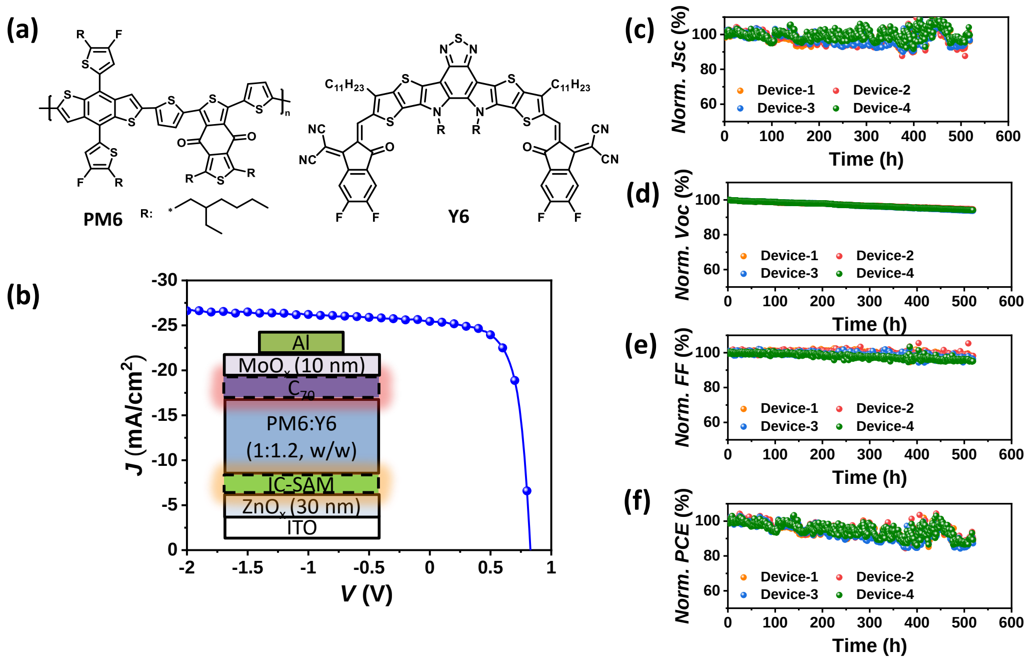


**Supplementary Figure 18.** (a) Molecular structural formulae of PM6 and Y6, (b) Current-density-voltage characteristics of fresh PM6:Y6 (1:1.2, w/w) devices. Inset, schematic of the device structure with $J_{SC}$ 25.59 ± 0.35 mA cm$^{-2}$; $V_{OC}$ 0.83 ± 0.01 V; fill factor 66 ± 2% and PCE 14.1 ± 0.6% before ageing, (c) Normalized $J_{SC}$, (d) $V_{OC}$, (e) *FF* and (d) *PCE* of a PM6:Y6 (1:1.2, w/w) OPV plotted vs. aging time under 1-sun simulated AM1.5G illumination with IC-SAM as a cathode buffer, $C_{70}$ as an anode buffer and 400 nm longpass UV-filter.

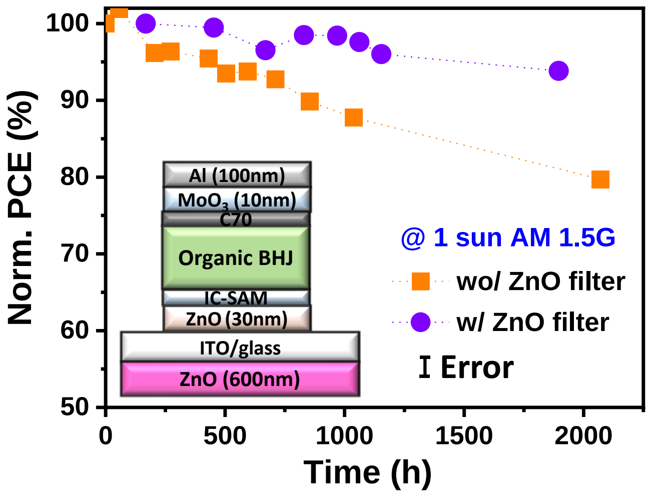


**Supplementary Figure 19.** Normalized PCE of the OPV integrated with ZnO UV filter and one without the UV filter plotted vs. aging time under 1-sun simulated AM1.5G illumination. (averaged for 4 devices). The error bars indicate the 1 s.d. uncertainty of each measurement.

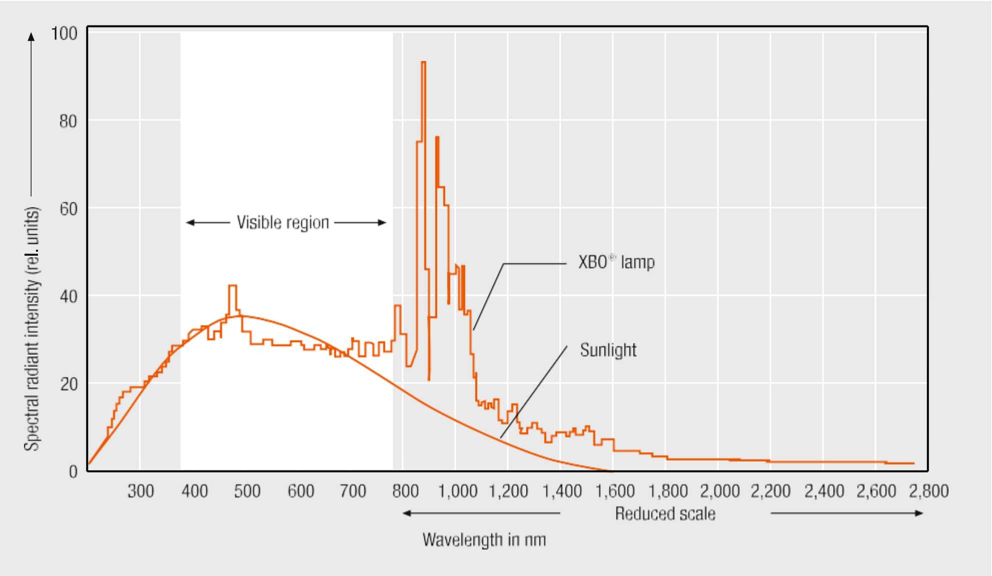


**Supplementary Figure 20.** The spectral distribution of radiant intensity of a typical XBO® Xenon lamp (1600 W/HS OFR XL) and a 6,200 K black body radiator. (About 6 % of the electric power consumed is emitted in the form of UV radiation below 380 nm, provide by vendor.)

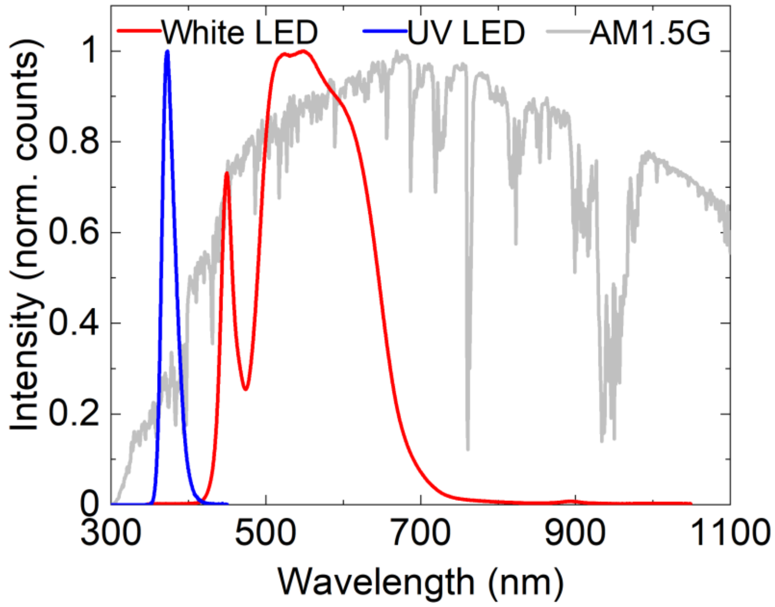


**Supplementary Figure 21.** The spectra of the white LEDs and UV LED, compared with the AM1.5G spectrum.

## References


1. Gasparini N*, et al.* (2017) Burn-in Free Nonfullerene-Based Organic Solar Cells. *Adv. Energy Mater.* 7(19):1700770.
2. Du X*, et al.* (2019) Efficient Polymer Solar Cells Based on Non-fullerene Acceptors with Potential Device Lifetime Approaching 10 Years. *Joule* 3(1):215-226.
3. Du X*, et al.* (2020) Unraveling the Microstructure-Related Device Stability for Polymer Solar Cells Based on Nonfullerene Small-Molecular Acceptors. *Adv. Mater.* 32(16):1908305.
4. Xie C*, et al.* (2018) Overcoming efficiency and stability limits in water-processing nanoparticular organic photovoltaics by minimizing microstructure defects. *Nat. Commun.* 9(1):5335.
5. Xu X*, et al.* (2020) Interface-enhanced organic solar cells with extrapolated T80 lifetimes of over 20 years. *Sci. Bull.* 65(3):208-216.
6. Liu H*, et al.* (2019) Boosting Organic–Metal Oxide Heterojunction via Conjugated Small Molecules for Efficient and Stable Nonfullerene Polymer Solar Cells. *Adv. Energy Mater.* 9(34):1900887.
7. Zhang M*, et al.* (2021) Single-layered organic photovoltaics with double cascading charge transport pathways: 18% efficiencies. *Nat. Commun.* 12(1):309.
8. Liao Q*, et al.* (2021) Highly Stable Organic Solar Cells Based on an Ultraviolet-Resistant Cathode Interfacial Layer. *CCS Chemistry* 3(4):1059-1069.
9. Lin Y*, et al.* (2020) Self-Assembled Monolayer Enables Hole Transport Layer-Free Organic Solar Cells with 18% Efficiency and Improved Operational Stability. *ACS Energy Lett.* 5(9):2935-2944.
10. Jiang Y*, et al.* (2019) Photocatalytic effect of ZnO on the stability of nonfullerene acceptors and its mitigation by SnO2 for nonfullerene organic solar cells. *Mater. Horiz.* 6(7):1438-1443.
11. Hu L*, et al.* (2021) Significant Enhancement of Illumination Stability of Nonfullerene Organic Solar Cells via an Aqueous Polyethylenimine Modification. *J. Phys. Chem. Lett.* 12(10):2607-2614.
12. Soultati A*, et al.* (2020) Suppressing the Photocatalytic Activity of Zinc Oxide Electron-Transport Layer in Nonfullerene Organic Solar Cells with a Pyrene-Bodipy Interlayer. *ACS Appl. Mater. Interfaces* 12(19):21961-21973.
13. Park S & Son HJ (2019) Intrinsic photo-degradation and mechanism of polymer solar cells: the crucial role of non-fullerene acceptors. *J. Mater. Chem. A* 7(45):25830-25837.
14. Ding K, *et al.* (2021) Photogeneration and the bulk quantum efficiency of organic photovoltaics. *Energy Environ. Sci.* 14 (3), 1584-1593.
